# Prognostic value of tumor‐infiltrating lymphocytes and PD‐L1 expression in esophageal squamous cell carcinoma

**DOI:** 10.1002/cam4.70179

**Published:** 2024-09-12

**Authors:** Jie Hu, Takeshi Toyozumi, Kentaro Murakami, Satoshi Endo, Yasunori Matsumoto, Ryota Otsuka, Tadashi Shiraishi, Shinichiro Iida, Hiroki Morishita, Tenshi Makiyama, Yuri Nishioka, Masaya Uesato, Koichi Hayano, Akira Nakano, Hisahiro Matsubara

**Affiliations:** ^1^ Department of Frontier Surgery Graduate School of Medicine, Chiba University Chiba Japan

**Keywords:** esophageal squamous cell carcinoma, PD‐L1, regulatory T cells, tumor microenvironment, tumor‐infiltrating lymphocytes

## Abstract

**Background:**

Tumor cells (TC) participate in tumor progression by altering the immune responses in the tumor microenvironment. However, the clinical relevance and prognostic effect of PD‐L1 expression and tumor‐infiltrating lymphocytes (TILs) in esophageal squamous cell carcinoma (ESCC) are unknown. The purpose of this study was to investigate the interactions and clinical significance of PD‐L1 expression and TILs in ESCC.

**Methods:**

Tissue specimens were collected from 126 patients with ESCC who underwent curative esophagectomy. Immunohistochemical analysis and multiplex immunofluorescence for CD4, CD8, CD25, FOXP3, and PD‐L1 in the tumor were used to identify multiple tumor‐infiltrating immune cells (TIIC), Tregs, and TC.

**Results:**

PD‐L1 was expressed in tumor cells (PD‐L1 TC). PD‐L1 TIIC and PD‐L1 TC affected the biological behavior of TC. The positive expression rate of PD‐L1 TC and CD8^+^ TILs was 27.8% (35/126) and 31.7% (40/126), respectively. Kaplan–Meier analysis showed that overall survival (OS) was significantly associated with decreased CD8^+^ TILs and PD‐L1 TC‐positive expression, which promote ESCC progression and metastasis.

**Conclusion:**

Tumor depth, CD8, and PD‐L1 TC were independent prognostic factors in ESCC, and a predictive nomogram with these three risk factors improved the accuracy of predicting OS in patients with ESCC after surgical resection. The conjoint analysis of multiple immune‐related factors is beneficial for stratifying patient survival risk.

## 1 INTRODUCTION

Esophageal cancer (EC) is among the most common malignant tumors of the gastrointestinal tract, ranking seventh in terms of incidence and sixth in mortality.^1^ Among EC cases, more than 90% are esophageal squamous cell carcinoma (ESCC), with a dismal survival outcome.^2^ Despite multidisciplinary therapeutic approaches including immune checkpoint inhibitors, the prognosis for patients with ESCC remains poor.^3^

The complex tumor microenvironment (TME) involves several tumor‐immune interactions, wherein continuous interactions between tumor cells (TC) and immune cells are a predominant feature that affects the immune response and contribute to tumor survival and aggressiveness, even altering patient prognosis.^4^, ^5^ Accumulating evidence suggests that PD‐L1 expressed on TC or immune cells affects the interplay between TC and tumor‐infiltrating lymphocytes (TILs) in the TME, attenuating the host anti‐tumor immune response.^6^, ^7^ Tumoral PD‐L1 expression status is closely correlated with the progression of the tumor and provides prognostic information.^8^, ^9^ PD‐L1 expression is closely related to survival in ESCC but its clinical significance remains controversial.^10^, ^11^

TILs are the most indispensable immune cell component in the anti‐tumor immune response in the TME.^12^, ^13^ Accumulation of CD8^+^ TILs in tumor tissue correlates with favorable clinical outcomes.^14^, ^15^ CD4, CD25, and FOXP3 are essential for the development, maturation and functioning of Tregs and provide a suitable microenvironment facilitating tumor escape.^16^, ^17^, ^18^ However, the reliability of FOXP3 as an exclusive biomarker for identifying Tregs is debatable.^19^, ^20^, ^21^ Therefore, it is necessary to comprehensively understand the expression of PD‐L1 and TILs in the tumor immune microenvironment of ESCC and their interdependence to explore more reliable prognostic immune factors to predict the prognosis of patients with ESCC.

In the present study, multiple TILs (CD4^+^ TILs, CD8^+^ TILs, CD25^+^ TILs, FOXP3^+^ TILs, CD4^+^CD25^+^FOXP3^+^ Tregs) and PD‐L1 expression were assessed in tissues specimens of patients with ESCC who underwent curative esophagectomy without any preoperative therapy to assess their clinicopathological characteristics and prognosis. Clinical prediction models were generated by integrating overall survival (OS)‐associated risk factors for ESCC, and the accuracy of the results was tested using calibration curve and time‐dependent receiver operating characteristic (time‐dependent ROC) curves.

## 2 MATERIALS AND METHODS

### 2.1 Patients and sample preparation

Specifically, 126 patients with pathologically confirmed ESCC who underwent radical esophagectomy at Chiba University Hospital were enrolled in our study (January 2001–April 2017), and 24 patients received adjuvant chemotherapy after surgery. The patients who underwent preoperative treatment were excluded. Tumor specimens were fixed in 10% formaldehyde solution, embedded in paraffin blocks, and cut into 4 μm‐thick serial sections to evaluate immune cell infiltration and PD‐L1 expression. The staging classification was assessed using the TNM system (UICC 8th edition).^22^ The median follow‐up time after surgery was 39.4 months (range 2–153 months). OS was defined as the period from the date of surgery to death. This study was approved by the Clinical Research Ethics Committee of Chiba University and informed consent was obtained from all patients to use their surgical specimens for research purposes.

### 2.2 Immunohistochemistry

Sections were deparaffinized and incubated with Antigen Retrieval Solution (HistoVT One, pH 7.0, Nacalai, Japan) at 95°C for 40 min. Endogenous peroxidase activity was blocked using Peroxidase‐Blocking Solution (Dako S2023, Japan) for 30 min. Serial sections were incubated with anti‐human CD8 mouse monoclonal antibody (clone: C8/144B; 1/100; Dako), anti‐human FOXP3 mouse monoclonal antibody (clone: 236A/E7; cat. no. ab20034; 1/100; Abcam), and anti‐human PD‐L1 rabbit monoclonal antibody (clone: 28–8; cat. no. ab205921; 1/500; Abcam) overnight at 4°C. Sections were washed with Tris‐buffered saline with tween (Dako S3006), and incubated with the corresponding secondary antibody at 37°C for 60 min. After washing, the sections were incubated with 3,3‐diaminobenzidine tetrahydrochloride substrate chromogen solution (DAB; Dako K3468) at room temperature for 90 s, 25 min, and 90 s. Subsequently, the sections were counterstained with hematoxylin for 1 min, dehydrated using ethanol, mounted, and examined under low‐power fields (original magnification ×40) using a Motic microscope (BA210E Upright) to confirm the tumor area and integral dye state. CD8 and FOXP3 were localized on the membranes and in the nuclei of TILs as yellowish‐brown and tan signals, respectively. PD‐L1 was predominantly present and localized to the membrane or cytoplasm of tumor and immune cells, which were observed as brown signals of differing intensities.

### 2.3 Evaluation of immunohistochemistry

**FIGURE 1:**
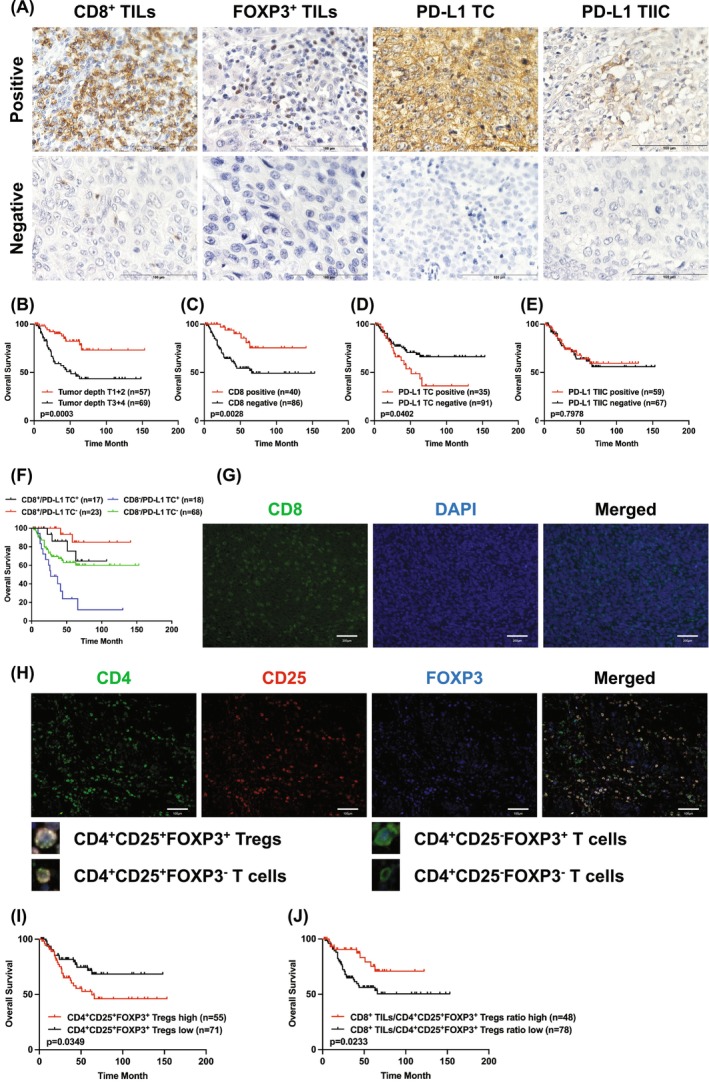
Tumor‐infiltrating lymphocytes, PD‐L1 expression and their associations with survival of patients with esophageal squamous cell carcinoma. (A) Representative immunohistochemistry images illustrating the infiltration of CD8^+^ TILs, FOXP3^+^ TILs, PD‐L1 TC, and PD‐L1 TIIC. (B) Kaplan–Meier analysis was used to determine the overall survival (OS) according to the prognostic effect of tumor depth (*p* = 0.0003), (C) CD8 (*p* = 0.0028), (D) PD‐L1 TC (*p* = 0.0402), and (E) PD‐L1 TIIC (*p* = 0.7978). (F) Kaplan–Meier curve for OS according to CD8/PD‐L1 TC status. (G) Representative immunofluorescence images of CD8 (green) and DAPI (blue) staining. (H) Immunofluorescence staining of CD4 (green), CD25 (red), and FOXP3 (blue) to label CD4^+^CD25^+^FOXP3^+^ Tregs, CD4^+^CD25^+^FOXP3^−^ T cells, CD4^+^CD25^−^FOXP3^+^ T cells, and CD4^+^CD25^−^FOXP3^−^ T cells, respectively. (I) Kaplan–Meier analysis was used to determine OS according to the prognostic effect of CD4^+^CD25^+^FOXP3^+^ Tregs (*p* = 0.0349) and (J) CD8^+^ TILs/CD4^+^CD25^+^FOXP3^+^ Tregs ratio (*p* = 0.0233). CD, cluster of differentiation; OS, overall survival; PD‐L1, programmed death ligand 1; TC, tumor cell; TIIC, tumor‐infiltrating immune cell; TILs, tumor‐infiltrating lymphocytes; Treg, regulatory T cells.

Five fields with the most adequately stained cells in the tumor center were captured and manually counted under a high‐power field (HPF) (magnification ×400) (Figure 1A) to assess CD8^+^ and FOXP3^+^ TILs. Each field included at least 200 immune or TC. The calculation was as follows: TILs (%) = positive immune cells/(immune cells + tumor cells) × 100.^23^ The final maximum value was the corresponding TILs percent. The optimal cutoff values for CD8^+^ and FOXP3^+^ TILs were 21% and 8%, respectively.

For TC, the evaluation was based on a previously reported method.^24^ The intensity of the staining was scored as follows: 0, negative; 1, weak; 2, moderate; and 3, strong. The proportion of PD‐L1 TC was scored as 0, <1%; 1, 1%–30%; 2, 31%–60%; and 3, 61%–100% (Figure S1). PD‐L1 TC was calculated as follows: PD‐L1 TC score (0–9) = proportion score (0–3) × intensity score (0–3). PD‐L1 TC expression was categorized as positive (≥4 score) or negative (<4 score).

PD‐L1 TIIC showed a homogeneous membrane staining pattern and scattered distribution at the tumor site. PD‐L1 TIICs included T cells, macrophages, dendritic cells, and other immune cells.^25^, ^26^, ^27^ Following previous studies,^28^, ^29^ PD‐L1 TIIC ≥1% was considered a positive expression.

Tissue sections were independently examined more than twice by two pathologists (H.J. and T.T.) who were blinded to the patient's clinical information. Necrotic areas were excluded from the observation field. In case of a discrepancy, the sections were reviewed by the pathologists together and the decision was made by consensus.

### 2.4 Immunofluorescence

Each section was deparaffinized and rehydrated using an ethanol gradient, followed by treatment with an antigen retrieval solution (HistoVT One, pH 7.0) at 95°C for 40 min. The sections were permeabilized using 0.1% Triton‐X‐100 for 10 min and non‐specific binding was blocked using the Peroxidase‐Blocking Solution (Dako S2023, Japan) for 30 min. The sections were incubated with a primary anti‐human CD25 monoclonal antibody (clone: EPR6452; cat. no. ab128955; 1/500; Abcam) overnight at 4°C. After several washes with phosphate‐buffered saline, the sections were incubated with secondary antibodies (Alexa Fluor 594; cat. no. A‐11012; 1/500; Invitrogen) in a moist dark chamber for 1 h. After several washes, the cells were incubated with a mixture of primary antibodies, including an anti‐CD4 monoclonal antibody (clone: EPR6855; cat. no. ab133616; 1/500; Abcam) and anti‐FOXP3 monoclonal antibody (clone: 236A/E7; cat. no. ab20034; 1/500; Abcam) for 1 h. The secondary antibody was a mixture of Goat anti‐Rabbit IgG (H + L) Cross‐Adsorbed Secondary Antibody Alexa Fluor 488 (cat. no. A‐11008; 1/500; Invitrogen) and Alexa Fluor 405 Goat Anti‐Mouse IgG H&L (cat. no. ab175660; 1/1000; Abcam, Cambridge, UK). An antibody against CD8 was used for double immunofluorescence staining. The specimens were incubated with an anti‐CD8 monoclonal antibody (clone: C8/144B; 1/100; Dako) overnight at 4°C, followed by incubation with a secondary antibody (Alexa Fluor 488; cat. no. A‐11001; 1:1000; Invitrogen) at room temperature for 1 h. DAPI Fluoromount‐G (cat. no. 0100–20, Southern Biotech) was used to mount the slides after staining. Multiplex immunofluorescence staining assessment was performed under a confocal laser scanning microscope KEYENCE BZ‐X700 (BIOREVO; KEYENCE Corp., Osaka, Japan) and the entire slide was scanned. Images were analyzed using the Image‐Pro Plus software (Media Cybernetics).

### 2.5 Evaluation of immunofluorescence

Tumor‐infiltrating T lymphocytes in each tissue specimen were observed at low‐power fields (original magnification ×100). Three or more of the most abundantly distributed areas in the tumor center were selected, and high‐power field (HPF) (original magnification ×400) images were obtained for exact quantification using Image‐Pro Plus software. The total number of cells was analyzed for CD4, CD8, CD25, and FOXP3 immunofluorescence staining intensity, and the maximum was recorded. A total of 89 cells/HPF, 111 cells/HPF, and 60 cells/HPF were defined as the optimal cut‐offs for CD4^+^ TILs, CD25^+^ TILs, and FOXP3^+^ TILs, respectively, and classified into high and low groups.

The following T cell phenotypes were quantified separately using the identical set of merged images: CD4^+^CD25^+^FOXP3^+^, CD4^+^CD25^+^FOXP3^−^, CD4^+^CD25^−^FOXP3^+^, and CD4^+^CD25^−^FOXP3^−^ (Figure 1H). CD4 (green signal) and CD25 (red signal) staining were detected on the T‐cell membrane, and FOXP3 (blue signal) was observed in the cell nucleus with a typical dotted staining pattern. T cells with the CD4^+^CD25^+^FOXP3^+^ phenotype were considered Tregs.^17^ The optimal cutoff values corresponding to the four T cell phenotypes were 80 cells/HPF, 2 cells/HPF, 1 cell/HPF, and 2 cells/HPF, respectively.

### 2.6 Statistical analysis

All statistical analyses were performed using GraphPad Prism 10.2.0, SPSS 29.0, and R software (http://www.r‐project.org/). Clinicopathological characteristics were analyzed using Chi‐Square and Fisher's exact tests. The optimal cutoff values of TIL markers and PD‐L1 TC expression were determined by ROC curve analysis. The correlation matrix for the markers was based on Spearman's rank correlation analysis. A two‐sided *t*‐test for unpaired samples was used to examine statistically significant differences between PD‐L1 and T‐cell phenotypes. The Kaplan–Meier curve was used to assess survival. Cox proportional hazards regression was used to identify the prognostic impact of the clinical variables and multiple potential biomarkers. All *p*‐values were two‐sided, and a *p*‐value <0.05 indicated a statistically significant result. Nomogram was constructed using the “rms” package in R (version 4.2.2). Calibration analyses were performed to examine the consistency between the risk predicted by the clinical prediction model and the actual risk of occurrence. Time‐dependent ROC analysis was performed to compare the ability of specific prognostic indicators to predict OS.

## 3 RESULTS

### 3.1 Clinical characteristics of the patients

In the present study cohort, 106 (84.1%) patients were males and 20 (15.9%) were females, with an average age of 66.6 years old (range 38–84). Sixty‐one (48.4%) patients were diagnosed with Stage I + II disease, and 65 (51.6%) were diagnosed with Stage III + IV disease. Fifty‐seven (45.2%) patients had T1 + 2, and 69 (54.8%) had T3 + 4. Twenty‐four patients (19.0%) underwent postoperative treatment; three patients underwent chemoradiotherapy with 5‐fluorouracil (5‐FU) and cisplatin (CDDP), 2 received 5‐FU only, 16 received 5‐fluorouracil and cisplatin (FP), and 3 received TS‐1. The clinicopathological characteristics of the patients are presented in Table 1.

**TABLE 1:** Correlation between CD8, PD‐L1 expression, and clinicopathological characteristics in patients with esophageal squamous cell carcinoma (*n* = 126).

	Total (*n*, %)	CD8 negative (*n* = 86)	CD8 positive (*n* = 40)	*p*‐value^a^	PD‐L1 TC negative (*n* = 91)	PD‐L1 TC positive (*n* = 35)	*p*‐value^a^	PD‐L1 TIIC negative (*n* = 67)	PD‐L1 TIIC positive (*n* = 59)	*p*‐value^a^
Gender				0.733			0.275			**0.013** ^b^
Male	106 (84.1%)	73	33		74	32		51	55	
Female	20 (15.9%)	13	7		17	3		16	4	
Age (years)				0.115			0.258			0.335
<66.6	57 (45.2%)	43	14		44	13		33	24	
>66.6	69 (54.8%)	43	26		47	22		34	35	
Tumor depth				0.674			**0.006** ^b^			0.804
T1 + 2	57 (45.2%)	40	17		48	9		31	26	
T3 + 4	69 (54.8%)	46	23		43	26		36	33	
Lymph node metastasis				0.081			0.287			0.452
N0	49 (38.9%)	29	20		38	11		24	25	
N1+	77 (61.1%)	57	20		53	24		43	34	
Stage				0.313			**0.049** ^b^			0.876
I + II	61 (48.4%)	39	22		49	12		32	29	
III + IV	65 (51.6%)	47	18		42	23		35	30	
Lymphatic invasion (Ly)				0.133			0.258			0.639
Negative	57 (45.2%)	35	22		44	13		29	28	
Positive	69 (54.8%)	51	18		47	22		38	31	
Venous invasion (V)				0.331			0.395			0.215
Negative	28 (22.2%)	17	11		22	6		12	16	
Positive	98 (77.8%)	69	29		69	29		55	43	
Postoperative treatment				**0.028** ^b^			0.091			0.914
Absent	102 (81.0%)	65	37		77	25		54	48	
Present	24 (19.0%)	21	3		14	10		13	11	

Abbreviations: PD‐L1, programmed cell death ligand 1; TC, tumor cell; TIIC, tumor‐infiltrating immune cell.

^a^ Statistical significance is determined using the Chi‐square test or Fisher's exact test.

^b^ The bold values in the table indicate that the *p*‐value is statistically significant.

### 3.2 TILs, PD‐L1 expression, and clinicopathological characteristics in ESCC

CD8^+^ TILs, PD‐L1 TC, and PD‐L1 TIICs were assessed (Figure 1A) and positive expressions were observed in 31.7% (40/126), 27.8% (35/126), and 46.8% (59/126) of patients, respectively. CD8^+^ TILs were inversely associated with postoperative treatment (*p* = 0.028). PD‐L1 TC levels correlated significantly with tumor depth and advanced stage. PD‐L1 TIIC was more common in male patients with ESCC (*p* = 0.013) (Table 1). The percentage of CD4^+^, CD25^+^, and FOXP3^+^ TILs was 37.3% (47/126), 39.7% (50/126), and 45.2% (57/126), respectively. CD4^+^ TILs infiltration was closely correlated with tumor depth (*p* = 0.020; Table S1). As shown in Table 2, PD‐L1 TC was correlated with CD8 (*p* = 0.0202), PD‐L1 TIIC (*p* < 0.0001), CD4 (*p* = 0.0174), and CD4^+^CD25^+^FOXP3^+^ Tregs (*p* = 0.0347). PD‐L1 TIICs were accompanied by an increased infiltration of CD8^+^ TILs (*p* = 0.0283) and CD4^+^ TILs (*p* = 0.0002).

**TABLE 2:** Correlation between PD‐L1 expression and T cell phenotypes in patients with esophageal squamous cell carcinoma (*n* = 126).

	PD‐L1 TC negative	PD‐L1 TC positive	*p*‐value^a^	PD‐L1 TIIC negative	PD‐L1 TIIC positive	*p‐*value^a^
Immunohistochemistry						
CD8 mean	17.02	20.83	**0.0202** ^b^	16.57	19.80	**0.0283** ^b^
FOXP3 mean	7.79	6.17	0.1553	6.78	7.98	0.2387
PD‐L1 TC mean	–	–	–	1.75	3.73	**<0.0001** ^b^
PD‐L1 TIIC mean	0.35	0.77	**<0.0001** ^b^	–	–	**–**
Immunofluorescence						
CD4 mean	95.09	133.30	**0.0174** ^b^	80.87	133.90	**0.0002** ^b^
CD25 mean	119.50	150.80	0.0807	125.90	130.80	0.7601
FOXP3 mean	66.80	73.23	0.6172	62.64	75.34	0.2703
CD4^+^CD25^+^FOXP3^+^ Tregs mean	96.41	133.30	**0.0347** ^b^	103.20	110.60	0.6414
CD4^+^CD25^+^FOXP3^−^ mean	11.09	8.00	0.5612	10.21	10.25	0.9924
CD4^+^CD25^−^FOXP3^+^ mean	0.98	0.77	0.8049	1.25	0.54	0.3424
CD4^+^CD25^−^FOXP3^−^ mean	1.31	0.11	0.5463	1.67	0.19	0.4026
Other						
CD8^+^TILs/CD4^+^CD25^+^FOXP3^+^ Tregs ratio mean	1.602	1.404	0.4525	1.359	1.760	0.0873

Abbreviations: CD, cluster of differentiation; FOXP3, forkhead box P3; PD‐L1, programmed cell death ligand 1; TC, tumor cell; TIIC, tumor‐infiltrating immune cell; TILs, tumor‐infiltrating lymphocytes; Tregs, regulatory T cells.

^a^ Statistical significance is determined by *t*‐test.

^b^ The bold values in the table indicate that the *p*‐value is statistically significant.

### 3.3 Correlation between tumor depth, CD8 ^+^ TILs, PD‐L1 expression, and prognosis in ESCC

Among the clinicopathological characteristics, the median survival of patients ESCC with T1 + 2 and T3 + 4 was 44.6 and 27.0 months, respectively (*p* = 0.0003, Figure 1B). The OS rate was significantly higher in patients with positive CD8 expression (5‐year OS: positive vs. negative: 80.9% vs. 54.4%, *p* = 0.0028, Figure 1C). Compared with PD‐L1 TC‐negative patients, the 5‐year postoperative survival rate of patients with positive expression was significantly lower (*p* = 0.0402, Figure 1D). We found no statistically significant difference between PD‐L1 TIIC‐positive and ‐negative patients (5‐year OS: positive vs. negative: 64.1% vs. 61.8%, Figure 1E).

According to the status of CD8 and PD‐L1 TC, patients were classified as CD8^+^/PD‐L1 TC^+^ (17/126, 13.5%), CD8^+^/PD‐L1 TC^−^ (23/126, 18.2%), CD8^−^/PD‐L1 TC^+^ (18/126, 14.3%) and CD8^−^/PD‐L1 TC^−^ (68/126, 54.0%) (Figure 1F and Table S2).

### 3.4 Relation between patient prognosis, T cell phenotypes, and clinicopathological characteristics

The results of multicolor immunofluorescence staining are shown in Figure 1G,H. CD8 and DAPI co‐labeled CD8^+^ T cells were used to evaluate CD8^+^ TILs. CD4, CD25, and FOXP3 co‐labeled T cell phenotypes were used to assess tumor‐infiltrating Tregs. High infiltration of CD4^+^CD25^+^FOXP3^+^ Tregs was observed in 43.7% (55/126) of patients. CD4^+^CD25^+^FOXP3^+^ Treg infiltration was correlated with lymph node metastasis (*p* = 0.047), stage (*p* = 0.017), and lymphatic invasion (*p* = 0.004, Table 3). Kaplan–Meier curves revealed that patients with high CD4^+^CD25^+^FOXP3^+^ Tregs had significantly decreased survival rate (5‐year OS: high vs. low: 52.8% vs. 71.9%, *p* = 0.0349, Figure 1I).

**TABLE 3:** Correlation between CD4^+^CD25^+^FOXP3^+^ Tregs, CD8^+^TILs/CD4^+^CD25^+^FOXP3^+^ Tregs ratio, and clinicopathological characteristics in patients with esophageal squamous cell carcinoma (*n* = 126).

	CD4^+^CD25^+^FOXP3^+^ Tregs			CD8^+^ TILs/CD4^+^CD25^+^FOXP3^+^ Tregs ratio		
	Low (*n* = 71)	High (*n* = 55)	*p*‐value^a^	Low (*n* = 78)	High (*n* = 48)	*p*‐value^a^
Gender			0.532			0.756
Male	61	45		65	41	
Female	10	10		13	7	
Age (years)			0.751			0.527
<66.6	33	24		37	20	
>66.6	38	31		41	28	
Tumor depth			0.078			**0.021** ^b^
T1 + 2	37	20		29	28	
T3 + 4	34	35		49	20	
Lymph node metastasis			**0.047** ^b^			**0.006** ^b^
N0	33	16		23	26	
N1+	38	39		55	22	
Stage			**0.017** ^b^			**0.034** ^b^
I + II	41	20		32	29	
III + IV	30	35		46	19	
Lymphatic invasion (Ly)			**0.004** ^b^			0.051
Negative	40	17		30	27	
Positive	31	38		48	21	
Venous invasion (V)			0.164			**0.019** ^b^
Negative	19	9		12	16	
Positive	52	46		66	32	
Postoperative treatment			**0.486**			**0.142**
Absent	59	43		60	42	
Present	12	12		18	6	

Abbreviations: CD, cluster of differentiation; TILs, tumor‐infiltrating lymphocytes; Tregs, regulatory T cells.

^a^ Statistical significance is determined using the Chi‐square test or Fisher's exact test.

^b^ The bold values in the table indicate that the *p*‐value is statistically significant.

### 3.5 CD8 ^+^ TILs/CD4 ^+^ CD25 ^+^ FOXP3 ^+^ Tregs ratio and clinical outcome in ESCC

Double and triple immunofluorescence staining confirmed CD8^+^ TILs and CD4^+^CD25^+^FOXP3^+^ Tregs, and the corresponding CD8^+^ TILs/CD4^+^CD25^+^FOXP3^+^ Treg ratios were calculated. The optimal cutoff value was 1.34 and patients were divided into high and low expression groups. CD8^+^ TILs/CD4^+^CD25^+^FOXP3^+^ Treg ratio was significantly correlated with T1 + 2 (28/57, 49.1%), lymph node metastasis (26/49, 53.1%), Stage I + II (29/61, 47.5%), and venous invasion (16/28, 57.1%) (Table 3).

CD8^+^ TILs/CD4^+^CD25^+^FOXP3^+^ Treg ratio was associated with survival of patients with ESCC (5‐year OS: high vs. low: 75.3% vs. 56.2%, *p* = 0.0233, Figure 1J).

### 3.6 Cox proportional hazard model for survival outcomes

Univariate analysis revealed that tumor depth, lymph node metastasis, stage, lymphatic invasion, venous invasion, infiltration of CD8 and FOXP3, PD‐L1 TC, CD25, CD4^+^CD25^+^FOXP3^+^ Tregs, and the CD8^+^ TILs/CD4^+^CD25^+^FOXP3^+^ Tregs ratio were significantly associated with OS.

In the multivariate analysis, tumor depth (HR: 0.303, 95% CI: 0.116–0.790, *p* = 0.015), CD8 infiltration (HR: 4.319, 95% CI: 1.386–13.454, *p* = 0.012), and PD‐L1 TC (HR: 0.455, 95% CI: 0.220–0.943, *p* = 0.034) were independent prognostic factors for ESCC (Table 4).

**TABLE 4:** Univariate and multivariate Cox regression analysis for overall survival of patients with esophageal squamous cell carcinoma (*n* = 126).

Characteristics	Univariate analysis		Multivariate analysis	
	*p*‐value	HR (95% CI)	*p*‐value	HR (95% CI)
Gender (female vs. male)	0.071	0.338 (0.104–1.095)	–	–
Age (<66.6 vs. >66.6)	0.546	1.208 (0.654–2.232)	–	–
Tumor depth (T1 + 2 vs. T3 + 4)	**0.001**	0.294 (0.144–0.600)	**0.015**	0.303 (0.116–0.790)
Lymph node metastasis (N0 vs. N1+)	**0.001**	0.234 (0.098–0.557)	0.184	0.380 (0.091–1.583)
Stage (I + II vs. III + IV)	**0.001**	0.289 (0.141–0.589)	0.530	1.568 (0.385–6.384)
Lymphatic invasion (negative vs. positive)	**0.003**	0.344 (0.168–0.702)	0.396	0.669 (0.265–1.691)
Venous invasion (negative vs. positive)	**0.010**	0.215 (0.066–0.697)	0.255	0.484 (0.138–1.691)
Immunohistochemistry				
CD8 infiltration (negative vs. positive)	**0.005**	3.452 (1.451–8.213)	**0.012**	4.319 (1.386–13.454)
FOXP3 infiltration (negative vs. positive)	**0.037**	2.051 (1.045–4.024)	0.872	0.904 (0.262–3.113)
PD‐L1 TC expression (negative vs. positive)	**0.044**	0.528 (0.283–0.984)	**0.034**	0.455 (0.220–0.943)
PD‐L1 TIIC expression (negative vs. positive)	0.799	1.084 (0.583–2.014)	–	–
Immunofluorescence				
CD4 infiltration (low vs. high)	0.053	2.075 (0.990–4.348)	–	–
CD25 infiltration (low vs. high)	**0.032**	0.508 (0.274–0.942)	0.258	0.538 (0.184–1.574)
FOXP3 infiltration (low vs. high)	**0.028**	2.096 (1.085–4.049)	0.313	1.901 (0.546–6.622)
CD4^+^CD25^+^FOXP3^+^ Tregs infiltration (low vs. high)	**0.039**	0.516 (0.275–0.967)	0.872	1.103 (0.337–3.612)
Other				
CD8^+^ TILs/CD4^+^CD25^+^FOXP3^+^ Tregs ratio (low vs. high)	**0.028**	2.295 (1.094–4.813)	0.664	0.801 (0.295–2.175)

Abbreviations: CD, cluster of differentiation; CI, confidence interval; FOXP3, forkhead box protein 3; HR, hazard ratio; PD‐L1, programmed death ligand 1; TC, tumor cell; TIIC, tumor‐infiltrating immune cell; TILs, tumor‐infiltrating lymphocytes; Tregs, regulatory T cells.

The bold values indicate that the *p*‐value is statistically significant.

### 3.7 Predictive nomogram for overall survival of patients with ESCC

**FIGURE 2:**
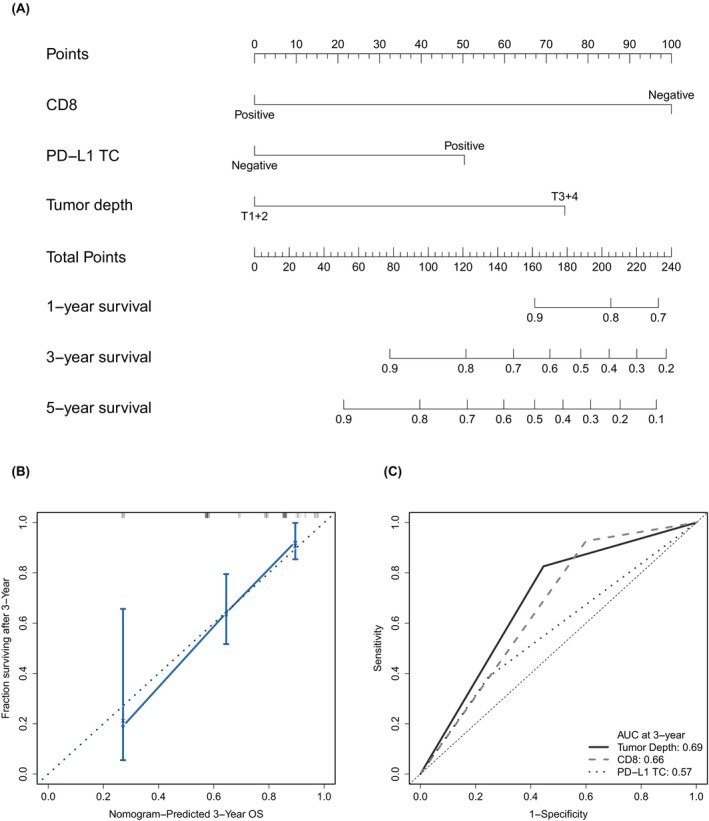
Prognostic nomogram to predict the overall survival (OS) of patients with esophageal squamous cell carcinoma. (A) A predictive nomogram for overall survival was constructed by combining proven independent prognostic factors with UICC tumor depth (T1 + 2, T3 + 4), CD8 expression (positive, negative), and PD‐L1 TC (positive, negative) expression. (B) The calibration plot for the nomogram to predict 3‐year survival and observed survival. (C) Time‐dependent receiver operating characteristic curves for nomogram, tumor depth, and CD8 and PD‐L1 TC expression to evaluate the 3‐year overall survival probability. *p*‐values were determined using the log‐rank test.

Based on the results of the multivariate analysis, tumor depth, CD8, and PD‐L1 TC levels were included in the nomogram prediction model to predict the 1‐, 3‐, and 5‐year survival probabilities (Figure 2A). The 3‐year calibration curves demonstrated a high consensus between the predicted and actual survival rates, with a C‐index of 0.756 (0.724–0.788), indicating that our predictive model was highly discriminative and accurate (Figure 2B). Time‐dependent ROC was used for validation, and the areas under the ROC curve (AUC) for tumor depth, CD8, and PD‐L1 TC levels were 0.69, 0.66, and 0.57, respectively (Figure 2C).

## 4 DISCUSSION

In the present study, we investigated CD4^+^ TILs, CD8^+^ TILs, CD25^+^ TILs, FOXP3^+^ TILs, CD4^+^CD25^+^FOXP3^+^ Tregs, and PD‐L1 expression status and their clinical significance in patients with ESCC. CD8^+^ TILs, PD‐L1 expression, and accumulation of CD4^+^CD25^+^FOXP3^+^ Tregs are involved in immune homeostasis, tumor proliferation, and metastasis. CD8 and PD‐L1 expression were independent prognostic immune‐related factors in ESCC.

The upregulation of PD‐L1 affects the development, progression, and prognosis of malignant tumors,^30^, ^31^ however, this finding remains controversial in ESCC. Guo et al.^32^ suggest that PD‐L1 expression contributes to patients with early‐stage cancer achieving a better clinical prognosis; in contrast, Yagi et al.^11^ demonstrated that high PD‐L1 expression was among high‐risk factors for patients with EC, and preoperative treatment tended to upregulate PD‐L1 expression. The bias may be due to differences in subjects, pre and postoperative treatments, antibodies, and evaluation methods.^10^, ^11^ In this regard, we excluded the tremendous impact of neoadjuvant chemotherapy and adjuvant therapy on TME and patient clinical outcomes and objectively evaluated PD‐L1 TC using standardized evaluation methods (staining intensity and proportion). The positive expression rate of intratumoral PD‐L1 in TC was 27.8%, which was closely associated with tumor depth, stage, and clinical outcomes. Compared with the negative PD‐L1 TC group, the positive group showed a 19.8% decrease in long‐term survival, suggesting that PD‐L1 TCs are associated with poor survival and participate in the development and progression of ESCC.

PD‐L1, apart from its expression in malignant TC, has been detected in tumor‐infiltrating immune cells. However, their clinical significance is not identical.^25^, ^27^ We evaluated intratumoral PD‐L1 TC and PD‐L1 TIIC in ESCC and found the association of only PD‐L1 TC with unfavorable clinical outcomes. Although Johnson et al.^26^ reported the dual roles of PD‐L1 in immune cells, the mechanism of PD‐L1 TIIC action in ESCC remains unknown. The crucial role of bidirectional PD‐L1 signaling in tumor immunity merits further investigation.

TILs, which mediate key antitumor immune responses, constitute a specific cell population in the TME. Intratumoral CD8^+^ T cells act as cytotoxic killer cells and eliminate TC. In the present study, the 5‐year survival rate of the CD8 positive group was higher than that of the negative group, along with serving as an independent favorable prognostic factor for ESCC. Unlike tumor eliminating CD8^+^ T cells, the clinical significance of CD4^+^ T cells is controversial.^33^, ^34^ Mumberg and Bos et al. reported that cytokines, such as IL‐2, IFN‐γ, and TNF‐α produced by activated CD4^+^ T cells can initiate and activate CD8^+^ T cells, and promote CD8^+^ T cells proliferation to inhibit tumor growth.^35^, ^36^ However, the ability of CD4^+^ TILs to directly alter the tumor‐host biological was not found in our study. This discrepancy may be due to the heterogeneity of the CD4^+^ T cells phenotype. Immunosuppressive Tregs differentiated from CD4^+^ T cells cannot be ignored. The aggregation of tumor‐infiltrating Tregs regulates immune homeostasis. Our findings revealed that the recruitment of CD4^+^CD25^+^FOXP3^+^ Tregs at the tumor site was associated with an advanced‐stage and lymph node metastasis, and up to 70.9% of patients with high CD4^+^CD25^+^FOXP3^+^ Tregs infiltration showed lymph node metastasis. CD4^+^CD25^+^FOXP3^+^ Tregs are associated with poor prognosis in patients with ESCC, suggesting that CD4^+^CD25^+^FOXP3^+^ Tregs are crucial for enhancing the invasive ability of TC and facilitating tumor escape. Furthermore, the status of immune homeostasis is a promising and valuable standard for identifying the significance of disease progression.^37^, ^38^ Our data showed that the CD8^+^ TILs/CD4^+^CD25^+^FOXP3^+^ Treg ratio was associated with improved OS and inversely correlated with tumor invasion and metastasis, suggesting that immune balance affects prognosis. However, it was not as dominant as CD8^+^ TILs or PD‐L1 TC in the multivariate analysis.

Nabeki et al.^39^ indicated that FoxP3+ Tregs are stimulated by tumor‐derived pro‐inflammatory cytokines, and elevated levels of FoxP3+ Tregs are significantly correlated with poor prognosis in ESCC. However, FOXP3‐expressing cells in tumor tissues, a functionally plastic cell population in vivo, confer unregulated properties to T cells.^40^, ^41^ This suggests that FOXP3 alone is insufficient as a specific marker of tumor‐infiltrating Tregs. This was confirmed in our study; opposite clinical results were obtained for Tregs labeled with FOXP3 alone and for Tregs co‐labeled with CD4 and CD25.

The effect of TILs or PD‐L1 on the prognosis of patients with ESCC was not a singular factor; cellular synergy interactions exert a more significant impact on the prognosis. Specific subpopulations of TIL components, infiltration numbers, and PD‐L1 expression in the TME are factors required for patient stratification. The 5‐year OS rate of the CD8^+^/PD‐L1 TC^−^ group patients was up to 84.9%, whereas it was only 24.1% in the CD8^−^/PD‐L1 TC^+^ group; lymph node metastasis was significantly higher in this group compared with the other three subgroups. This is consistent with the theory that TILs/PD‐L1 are involved in mediating adaptive immune resistance in tumor immunity.^42^ These results on the CD8/PD‐L1 TC status may be more beneficial for patient risk assessment.

In summary, as neoadjuvant therapy and immunotherapy gradually replace surgery as the first‐line treatment, it has become more difficult to obtain tumor samples that are unaffected by any treatment. Our results clarify the prognostic value of tumor depth, CD8^+^ TILs and PD‐L1 TC in patients with preoperatively untreated ESCC. Elucidating the immune mechanism of CD8^+^ TILs/PD‐L1 TC and CD8^+^ TILs/CD4^+^CD25^+^FOXP3^+^ Treg ratios may provide strategies for developing immunotherapy for ESCC.

## AUTHOR CONTRIBUTIONS

**Jie Hu:** Conceptualization (equal); data curation (equal); formal analysis (equal); investigation (equal); methodology (equal); resources (equal); software (equal); visualization (equal); writing – original draft (equal). **Takeshi Toyozumi:** Conceptualization (equal); data curation (equal); formal analysis (equal); funding acquisition (equal); investigation (equal); methodology (equal); project administration (equal); resources (equal); software (equal); supervision (equal); validation (equal); visualization (equal); writing – original draft (equal). **Kentaro Murakami:** Conceptualization (equal); data curation (equal); project administration (equal). **Satoshi Endo:** Conceptualization (equal); data curation (equal); project administration (equal). **Yasunori Matsumoto:** Conceptualization (equal); data curation (equal); project administration (equal). **Ryota Otsuka:** Conceptualization (equal); data curation (equal); project administration (equal). **Tadashi Shiraishi:** Conceptualization (equal). **Shinichiro Iida:** Conceptualization (equal). **Hiroki Morishita:** Conceptualization (equal). **Tenshi Makiyama:** Conceptualization (equal). **Yuri Nishioka:** Conceptualization (equal). **Masaya Uesato:** Conceptualization (equal); data curation (equal); project administration (equal). **Koichi Hayano:** Conceptualization (equal); data curation (equal); project administration (equal). **Akira Nakano:** Conceptualization (equal); data curation (equal); project administration (equal). **Hisahiro Matsubara:** Conceptualization (equal); funding acquisition (equal); project administration (equal); resources (equal); supervision (equal); validation (equal).

## FUNDING INFORMATION

The authors have received no funding support for this study.

## CONFLICT OF INTEREST STATEMENT

The authors declare no conflicts of interest.

## ETHICS STATEMENT

Approval of the Research Protocol by an Institutional Review Board: This study was approved by the Clinical Research Ethics Committee of Chiba University on 03 December 2019 (No. 3597).

## INFORMED CONSENT

Informed consent was obtained from all patients to use the surgical specimens for research purposes.

## Supporting information


Data S1.

## Data Availability

The data supporting the findings of this study are available from the corresponding author upon request.

## References

[1] Sung H , Ferlay J , Siegel RL , et al. Global cancer statistics 2020: GLOBOCAN estimates of incidence and mortality worldwide for 36 cancers in 185 countries. CA Cancer J Clin. 2021;71:209‐249. 33538338 10.3322/caac.21660

[2] Smyth EC , Lagergren J , Fitzgerald RC , et al. Oesophageal cancer. Nat Rev Dis Prim. 2017;3:17048. 28748917 10.1038/nrdp.2017.48 PMC6168059

[3] Kudo T , Hamamoto Y , Kato K , et al. Nivolumab treatment for oesophageal squamous‐cell carcinoma: an open‐label, multicentre, phase 2 trial. Lancet Oncol. 2017;18:631‐639. 28314688 10.1016/S1470-2045(17)30181-X

[4] Chen DS , Mellman I . Oncology meets immunology: the cancer‐immunity cycle. Immunity. 2013;39:1‐10. 23890059 10.1016/j.immuni.2013.07.012

[5] Anderson NM , Simon MC . The tumor microenvironment. Curr Biol. 2020;30:R921. 32810447 10.1016/j.cub.2020.06.081 PMC8194051

[6] Pardoll DM . The blockade of immune checkpoints in cancer immunotherapy. Nat Rev Cancer. 2012;12:252‐264. 22437870 10.1038/nrc3239 PMC4856023

[7] Wang Y , Wang H , Yao H , Li C , Fang JY , Xu J . Regulation of PD‐L1: emerging routes for targeting tumor immune evasion. Front Pharmacol. 2018;9:536. 29910728 10.3389/fphar.2018.00536 PMC5992436

[8] Muenst S , Schaerli AR , Gao F , et al. Expression of programmed death ligand 1 (PD‐L1) is associated with poor prognosis in human breast cancer. Breast Cancer Res Treat. 2014;146:15‐24. 24842267 10.1007/s10549-014-2988-5 PMC4180714

[9] Li C , Li C , Zhi C , et al. Clinical significance of PD‐L1 expression in serum‐derived exosomes in NSCLC patients. J Transl Med. 2019;17:355. 31665020 10.1186/s12967-019-2101-2 PMC6820965

[10] Tsutsumi S , Saeki H , Nakashima Y , et al. Programmed death‐ligand 1 expression at tumor invasive front is associated with epithelial‐mesenchymal transition and poor prognosis in esophageal squamous cell carcinoma. Cancer Sci. 2017;108:1119‐1127. 28294486 10.1111/cas.13237 PMC5480087

[11] Yagi T , Baba Y , Ishimoto T , et al. PD‐L1 expression, tumor‐infiltrating lymphocytes, and clinical outcome in patients with surgically resected esophageal cancer. Ann Surg. 2019;269:471‐478. 29206673 10.1097/SLA.0000000000002616

[12] Pozzesi N , Fierabracci A , Liberati AM , et al. Role of caspase‐8 in thymus function. Cell Death Differ. 2014;21:226‐233. 24270406 10.1038/cdd.2013.166 PMC3890959

[13] Robey E , Fowlkes BJ . Selective events in T cell development. Annu Rev Immunol. 1994;12:675‐705. 8011294 10.1146/annurev.iy.12.040194.003331

[14] Lee JS , Won HS , Sun S , Hong JH , Ko YH . Prognostic role of tumor‐infiltrating lymphocytes in gastric cancer: a systematic review and meta‐analysis. Medicine (Baltimore). 2018;97:e11769. 30095632 10.1097/MD.0000000000011769 PMC6133557

[15] Idos GE , Kwok J , Bonthala N , Kysh L , Gruber SB , Qu C . The prognostic implications of tumor infiltrating lymphocytes in colorectal cancer: a systematic review and meta‐analysis. Sci Rep. 2020;10:3360. 32099066 10.1038/s41598-020-60255-4 PMC7042281

[16] Sakaguchi S , Miyara M , Costantino CM , Hafler DA . FOXP3^+^ regulatory T cells in the human immune system. Nat Rev Immunol. 2010;10:490‐500. 20559327 10.1038/nri2785

[17] Sakaguchi S , Ono M , Setoguchi R , et al. FOXP3^+^ CD25^+^ CD4^+^ natural regulatory T cells in dominant self‐tolerance and autoimmune disease. Immunol Rev. 2006;212:8‐27. 16903903 10.1111/j.0105-2896.2006.00427.x

[18] Hori S , Nomura T , Sakaguchi S . Control of regulatory T cell development by the transcription factor FOXP3. Science. 2003;299:1057‐1061. 28115586

[19] Seminerio I , Descamps G , Dupont S , et al. Infiltration of FOXP3^+^ regulatory T cells is a strong and independent prognostic factor in head and neck squamous cell carcinoma. Cancers (Basel). 2019;11:227. 30781400 10.3390/cancers11020227 PMC6406934

[20] Brouwer T , Ijsselsteijn M , Oosting J , et al. A paradoxical role for regulatory T cells in the tumor microenvironment of pancreatic cancer. Cancers (Basel). 2022;14:3862. 36010856 10.3390/cancers14163862 PMC9405872

[21] Ladoire S , Martin F , Ghiringhelli F . Prognostic role of FOXP3^+^ regulatory T cells infiltrating human carcinomas: the paradox of colorectal cancer. Cancer Immunol Immunother. 2011;60:909‐918. 21644034 10.1007/s00262-011-1046-y PMC11028605

[22] Rice TW , Patil DT , Blackstone EH . 8th edition AJCC/UICC staging of cancers of the esophagus and esophagogastric junction: application to clinical practice. Ann Cardiothorac Surg. 2017;6:119‐130. 28447000 10.21037/acs.2017.03.14 PMC5387145

[23] Acs B , Ahmed FS , Gupta S , et al. An open source automated tumor infiltrating lymphocyte algorithm for prognosis in melanoma. Nat Commun. 2019;10:5440. 31784511 10.1038/s41467-019-13043-2 PMC6884485

[24] Kim DH , Kim H , Choi YJ , et al. Exosomal PD‐L1 promotes tumor growth through immune escape in non‐small cell lung cancer. Exp Mol Med. 2019;51:1‐13. 10.1038/s12276-019-0295-2 PMC6802663 31399559

[25] Diskin B , Adam S , Cassini MF , et al. PD‐L1 engagement on T cells promotes self‐tolerance and suppression of neighboring macrophages and effector T cells in cancer. Nat Immunol. 2020;21:442‐454. 32152508 10.1038/s41590-020-0620-x

[26] Johnson RMG , Wen T , Dong H . Bidirectional signals of PD‐L1 in T cells that fraternize with cancer cells. Nat Immunol. 2020;21:365‐366. 32152507 10.1038/s41590-020-0599-3

[27] Liu Y , Zugazagoitia J , Ahmed FS , et al. Immune cell PD‐L1 colocalizes with macrophages and is associated with outcome in PD‐1 pathway blockade therapy. Clin Cancer Res. 2020;26:970‐977. 31615933 10.1158/1078-0432.CCR-19-1040 PMC7024671

[28] Sumitomo R , Hirai T , Fujita M , Murakami H , Otake Y , Huang CL . PD‐L1 expression on tumor‐infiltrating immune cells is highly associated with M2 TAM and aggressive malignant potential in patients with resected non‐small cell lung cancer. Lung Cancer. 2019;136:136‐144. 31499335 10.1016/j.lungcan.2019.08.023

[29] Fehrenbacher L , Spira A , Ballinger M , et al. Atezolizumab versus docetaxel for patients with previously treated non‐small‐cell lung cancer (POPLAR): a multicentre, open‐label, phase 2 randomised controlled trial. Lancet. 2016;387:1837‐1846. 26970723 10.1016/S0140-6736(16)00587-0

[30] Fumet JD , Richard C , Ledys F , et al. Prognostic and predictive role of CD8 and PD‐L1 determination in lung tumor tissue of patients under anti‐PD‐1 therapy. Br J Cancer. 2018;119:950‐960. 30318514 10.1038/s41416-018-0220-9 PMC6203820

[31] Guo W , Wang P , Li N , et al. Prognostic value of PD‐L1 in esophageal squamous cell carcinoma: a meta‐analysis. Oncotarget. 2018;9:13920‐13933. 29568405 10.18632/oncotarget.23810 PMC5862626

[32] Guo W , Zhang F , Shao F , et al. PD‐L1 expression on tumor cells associated with favorable prognosis in surgically resected esophageal squamous cell carcinoma. Hum Pathol. 2019;84:291‐298. 30296523 10.1016/j.humpath.2018.09.014

[33] Bromwich EJ , McArdle PA , Canna K , et al. The relationship between T‐lymphocyte infiltration, stage, tumour grade and survival in patients undergoing curative surgery for renal cell cancer. Br J Cancer. 2003;89:1906‐1908. 14612901 10.1038/sj.bjc.6601400 PMC2394435

[34] Hiraoka K , Miyamoto M , Cho Y , et al. Concurrent infiltration by CD8^+^ T cells and CD4^+^ T cells is a favourable prognostic factor in non‐small‐cell lung carcinoma. Br J Cancer. 2006;94:275‐280. 16421594 10.1038/sj.bjc.6602934 PMC2361103

[35] Mumberg D , Monach PA , Wanderling S , et al. CD4^+^ T cells eliminate MHC class II‐negative cancer cells in vivo by indirect effects of IFN‐gamma. Proc Natl Acad Sci USA. 1999;96:8633‐8638. 10411927 10.1073/pnas.96.15.8633 PMC17568

[36] Bos R , Sherman LA . CD4^+^ T‐cell help in the tumor milieu is required for recruitment and cytolytic function of CD8^+^ T lymphocytes. Cancer Res. 2010;70:8368‐8377. 20940398 10.1158/0008-5472.CAN-10-1322 PMC2970736

[37] Tavares MC , Sampaio CD , Lima GE , et al. A high CD8 to FOXP3 ratio in the tumor stroma and expression of PTEN in tumor cells are associated with improved survival in non‐metastatic triple‐negative breast carcinoma. BMC Cancer. 2021;21:901. 34362334 10.1186/s12885-021-08636-4 PMC8343973

[38] Suzuki H , Chikazawa N , Tasaka T , et al. Intratumoral CD8^+^T/FOXP3^+^ cell ratio is a predictive marker for survival in patients with colorectal cancer. Cancer Immunol Immunother. 2010;59:653‐661. 19908042 10.1007/s00262-009-0781-9 PMC11030791

[39] Nabeki B , Ishigami S , Uchikado Y , et al. Interleukin‐32 expression and Treg infiltration in esophageal squamous cell carcinoma. Anticancer Res. 2015;35:2941‐2947. 25964580

[40] Komatsu N , Mariotti‐Ferrandiz ME , Wang Y , Malissen B , Waldmann H , Hori S . Heterogeneity of natural FOXP3^+^ T cells: a committed regulatory T‐cell lineage and an uncommitted minor population retaining plasticity. Proc Natl Acad Sci USA. 2009;106:1903‐1908. 19174509 10.1073/pnas.0811556106 PMC2644136

[41] Wing JB , Tanaka A , Sakaguchi S . Human FOXP3^+^ regulatory T cell heterogeneity and function in autoimmunity and cancer. Immunity. 2019;50:302‐316. 30784578 10.1016/j.immuni.2019.01.020

[42] Teng MW , Ngiow SF , Ribas A , Smyth MJ . Classifying cancers based on T‐cell infiltration and PD‐L1. Cancer Res. 2015;75:2139‐2145. 25977340 10.1158/0008-5472.CAN-15-0255 PMC4452411

