# Supplementary material for: Prognostic value of tumor‐infiltrating lymphocytes and PD‐L1 expression in esophageal squamous cell carcinoma
Source: Cancer Med. 2024 Sep 12;13(17):e70179. doi: 10.1002/cam4.70179 (PMC11391568; doi:10.1002/cam4.70179)
Supplement: Supplementary file 1 — Data S1. [file CAM4-13-e70179-s001.docx]

**Supporting information**

**Title:**

Prognostic value of tumor-infiltrating lymphocytes and PD-L1 expression in esophageal squamous cell carcinoma

**Authors’ names:**

Jie Hu, Takeshi Toyozumi, Kentaro Murakami, Satoshi Endo, Yasunori Matsumoto, Ryota Otsuka, Tadashi Shiraishi, Shinichiro Iida, Hiroki Morishita, Tenshi Makiyama, Yuri Nishioka, Masaya Uesato, Koichi Hayano, Akira Nakano, Hisahiro Matsubara

**Authors’ affiliations:**

Department of Frontier Surgery, Graduate School of Medicine, Chiba University, Chiba, Japan

**Corresponding author:**

Takeshi Toyozumi MD, PhD

Department of Frontier Surgery, Graduate School of Medicine, Chiba University

1-8-1 Inohana, Chuo-ku, Chiba, 260-8677, Chiba, Japan

Phone number: +81-43-222-7171

E-mail: t_toyozumi@chiba-u.jp

**Table of Contents:**

-Supplementary Figure 1 p.3

-Supplementary Figure 2 p.4

-Supplementary Figure 3 p.5

-Supplementary Table 1 p.6

-Supplementary Table 2 p.7

**Supplementary Figure 1. *Evaluation of PD-L1 TC.*** The intensity of staining was scored as follows: **(A)** 0, negative, total absence of staining. **(B)** 1, weak staining. **(C)** 2, moderate staining. **(D)** 3, strong staining (original magnification x400). The proportion of PD-L1 positive cells expressed in the tumor was scored as **(E)** 0, <1%. **(F)** 1, 1-30%. **(G)** 2, 31-60%. **(H)** 3, 61-100% (original magnification x100). PD-L1, programmed death ligand 1; TC, tumor cells.

**Supplementary Figure 2. *Kaplan-Meier curve for overall survival (OS) based on the status of the infiltration of tumor-infiltrating lymphocytes (TILs) in patients with ESCC.*** Kaplan-Meier curve for OS according to the infiltrating status of **(A)** CD4^+^ TILs (p=0.0473), **(B)** CD25^+^ TILs (p=0.0279), **(C)** FOXP3^+^ TILs (p=0.0239), **(D)** CD4^+^CD25^+^FOXP3^-^ T cells (p=0.1692), **(E)** CD4^+^CD25^-^FOXP3^+^ T cells (p=0.2986), and **(F)** CD4^+^CD25^-^FOXP3^-^ T cells (p=0.5842).


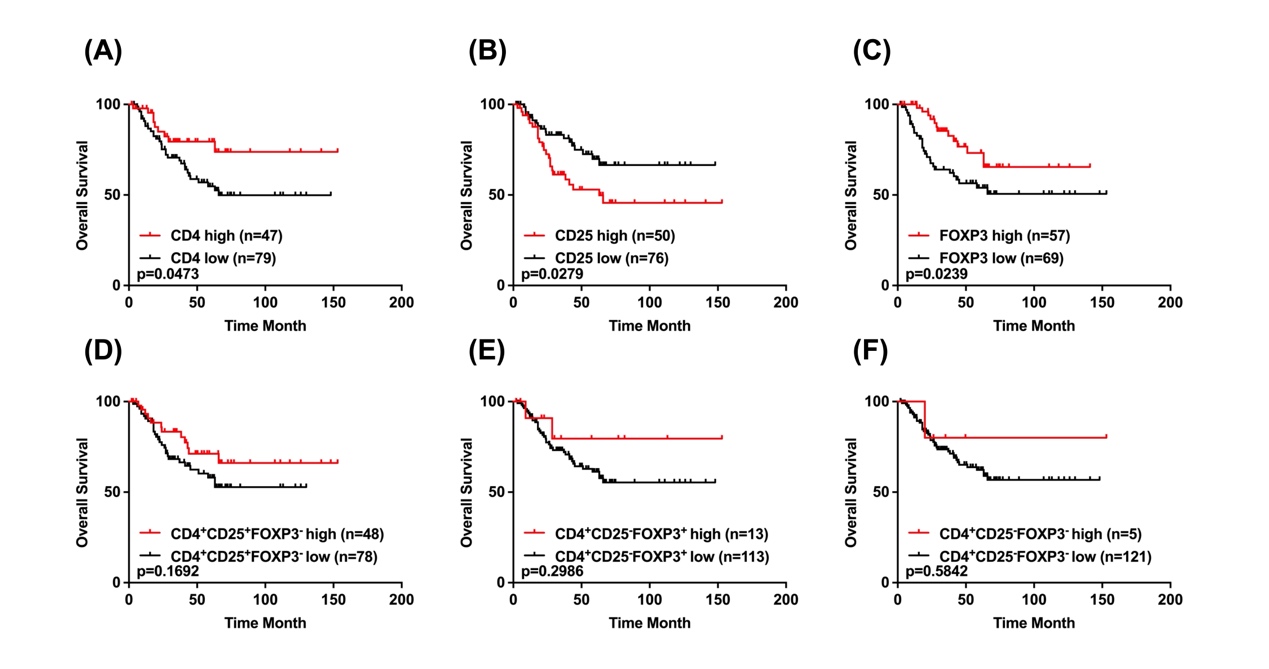


**Supplementary Figure S3. *Kaplan-Meier curve for overall survival (OS) based on the surgical alone and postoperative treatment groups of patients with ESCC.*** The 5-year OS rates were 68.0% and 43.3% after surgery alone and after postoperative surgery in our cohort, respectively.

**
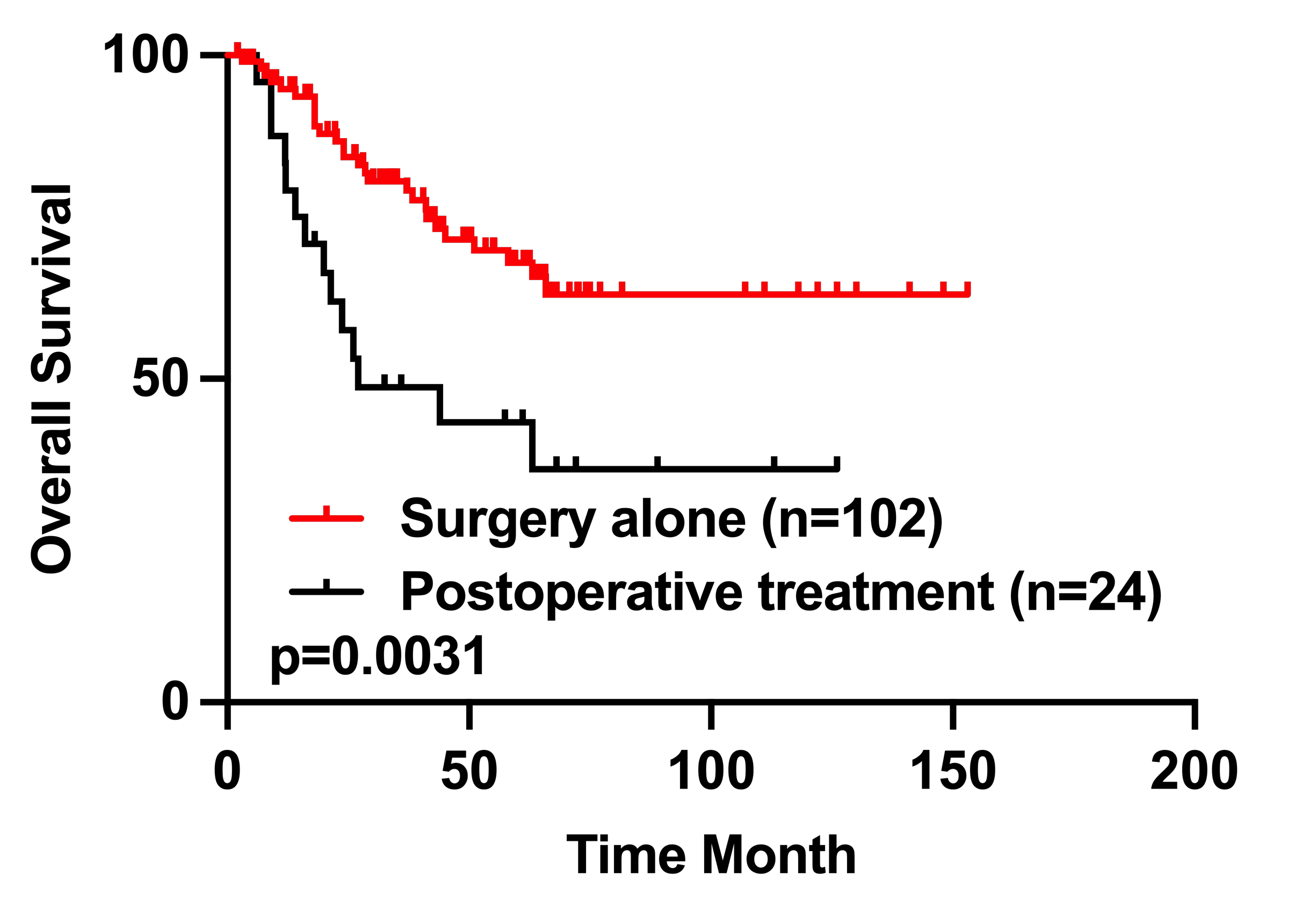
**

**Supplementary Table 1. *Correlation between tumor-infiltrating lymphocyte (TILs) and clinicopathological characteristics.***

|  | **CD4^+^ TILs** | | |  | **CD25^+^ TILs** | | |  | **FOXP3^+^ TILs** | | |
| --- | --- | --- | --- | --- | --- | --- | --- | --- | --- | --- | --- |
|  | **Low (n=79)** | **High (n=47)** | **p value^a^** |  | **Low (n=76)** | **High (n=50)** | **p value^a^** |  | **Low (n=69)** | **High (n=57)** | **p value^a^** |
| **Gender** |  |  | 0.438 |  |  |  | 0.304 |  |  |  | 0.981 |
| Male | 68 | 38 |  |  | 66 | 40 |  |  | 58 | 48 |  |
| Female | 11 | 9 |  |  | 10 | 10 |  |  | 11 | 9 |  |
| **Age (years)** |  |  | 0.227 |  |  |  | 0.821 |  |  |  | 0.778 |
| <66.6 | 39 | 18 |  |  | 35 | 22 |  |  | 32 | 25 |  |
| >66.6 | 40 | 29 |  |  | 41 | 28 |  |  | 37 | 32 |  |
| **Tumor depth** |  |  | **0.020^b^** |  |  |  | 0.185 |  |  |  | 0.130 |
| T1+2 | 42 | 15 |  |  | 38 | 19 |  |  | 27 | 30 |  |
| T3+4 | 37 | 32 |  |  | 38 | 31 |  |  | 42 | 27 |  |
| **Lymph node metastasis** |  |  | 0.785 |  |  |  | 0.097 |  |  |  | 0.298 |
| N0 | 30 | 19 |  |  | 34 | 15 |  |  | 24 | 25 |  |
| N1+ | 49 | 28 |  |  | 42 | 35 |  |  | 45 | 32 |  |
| **Stage** |  |  | 0.646 |  |  |  | 0.058 |  |  |  | 0.885 |
| I+II | 37 | 24 |  |  | 42 | 19 |  |  | 33 | 28 |  |
| III+IV | 42 | 23 |  |  | 34 | 31 |  |  | 36 | 29 |  |
| **Lymphatic invasion (Ly)** |  |  | 0.785 |  |  |  | 0.091 |  |  |  | 0.778 |
| Negative | 35 | 22 |  |  | 39 | 18 |  |  | 32 | 25 |  |
| Positive | 44 | 25 |  |  | 37 | 32 |  |  | 37 | 32 |  |
| **Venous invasion (V)** |  |  | 0.522 |  |  |  | 0.626 |  |  |  | 0.062 |
| Negative | 19 | 9 |  |  | 18 | 10 |  |  | 11 | 17 |  |
| Positive | 60 | 38 |  |  | 58 | 40 |  |  | 58 | 40 |  |

CD cluster of differentiation, FOXP3 forkhead box P3.

^a^ Statistical significance is determined using the Chi-square test or Fisher's exact test.

^b^ The bold values in the table indicate that the p-value is statistically significant.

**Supplementary Table 2. *Correlation between CD8/PD-L1 TC expression and clinicopathological characteristics.***

|  | **CD8^+^/PD-L1 TC^+^**  **(n=17)** | **CD8^+^/PD-L1 TC^-^**  **(n=23)** | **CD8^-^/PD-L1 TC^+^**  **(n=18)** | **CD8^-^/PD-L1 TC^-^**  **(n=68)** | ***p* value^a^** |
| --- | --- | --- | --- | --- | --- |
| **Gender** |  |  |  |  | 0.493 |
| Male | 15 | 18 | 17 | 56 |  |
| Female | 2 | 5 | 1 | 12 |  |
| **Age (years)** |  |  |  |  | 0.095 |
| <66.6 | 3 | 11 | 10 | 33 |  |
| >66.6 | 14 | 12 | 8 | 35 |  |
| **Tumor depth** |  |  |  |  | **0.027^b^** |
| T1+2 | 3 | 14 | 6 | 34 |  |
| T3+4 | 14 | 9 | 12 | 34 |  |
| **Lymph node metastasis** |  |  |  |  | 0.152 |
| N0 | 7 | 13 | 4 | 25 |  |
| N1+ | 10 | 10 | 14 | 43 |  |
| **Stage** |  |  |  |  | 0.107 |
| I+II | 7 | 15 | 5 | 34 |  |
| III+IV | 10 | 8 | 13 | 34 |  |
| **Lymphatic invasion (Ly)** |  |  |  |  | 0.142 |
| Negative | 9 | 13 | 4 | 31 |  |
| Positive | 8 | 10 | 14 | 37 |  |
| **Venous invasion (V)** |  |  |  |  | 0.435 |
| Negative | 3 | 8 | 3 | 14 |  |
| Positive | 14 | 15 | 15 | 54 |  |

CD cluster of differentiation, FOXP3 forkhead box protein 3, PD-L1 programmed death ligand 1, TC tumor cell.

^a^ Statistical significance is determined using the Chi-square test or Fisher's exact test.

^b^ The bold values in the table indicate that the p-value is statistically significant.
